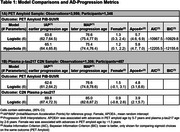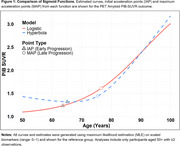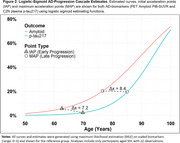# Estimating Early Biomarker Accelerations in AD‐progression: Modelling the Jack Curves

**DOI:** 10.1002/alz70861_108869

**Published:** 2025-12-23

**Authors:** Hunter C Sylvester, Murat Bilgel, Jeannette Simino, Clifford R. Jack, Petrice M Cogswell, Ronald Petersen, David S. Knopman, Prashanthi Vemuri, Jonathan Graff‐Radford, Thomas H. Mosley, Gwen B Windham, Michael E. Griswold

**Affiliations:** ^1^ University of Mississippi Medical Center, The MIND Center, Jackson, MS USA; ^2^ Laboratory of Behavioral Neuroscience, National Institute on Aging Intramural Research Program, National Institutes of Health, Baltimore, MD USA; ^3^ University of Mississippi Medical Center, Jackson, MS USA; ^4^ Department of Radiology, Mayo Clinic, Rochester, MN USA; ^5^ Department of Neurology, Mayo Clinic, Rochester, MN USA

## Abstract

**Background:**

Alzheimer’s Disease (AD) progression is conceptualized as a cascade of sigmoidal (S‐shaped) curves, with biomarkers shifting sequentially from normal to abnormal states. Estimating these curves requires sophisticated modeling, with challenges including (1) selecting appropriate sigmoidal‐type functions, (2) achieving algorithmic convergence, and (3) producing clinically useful parameters. We compare commonly used sigmoidal‐functions and effects on novel estimates of early and late AD‐biomarker progression ages.

**Method:**

Using 3,999 Amyloid PET (PiB SUVR) measurements from 1,367 Mayo Clinic Study of Aging participants, we compared model fits (AIC, BIC) between logistic and hyperbolic functions using Non‐Linear Mixed Effect (NLME) models with subject‐specific age‐shifts. We defined novel “initial” and “maximum” acceleration points (IAP and MAP) to compare clinically “earlier” and “later” AD‐progression ages between sigmoidal‐curves. The MAP uses the maximum second‐derivative point, reflecting the age of fastest acceleration in biomarker accumulation. The IAP is defined as the first point where the second derivative of the sigmoidal‐curve exceeds a clinical threshold (here, 5% velocity increase per year), indicating onset age of early accelerated biomarker accumulation. Estimates for C_2_N plasma *p* ‐tau217 were also attempted (1,306 measurements on 457 participants). Models adjusted for sex and APOE4‐positivity.

**Result:**

In the larger PET sample, the hyperbola had lower AIC/BIC (better fit), but showed a decreasing early PiB‐SUVR trend (Table 1A, Figure 1). Ages of late MAP were comparable, as were sex and APOE4+ related PiB‐SUVR‐curve shifts. Age of early AD‐onset was estimated 1.5 years earlier for logistic (IAP=63.6) versus hyperbola (IAP=65.1) functions. The logistic function always converged, while the hyperbolic function did not converge for *p* ‐tau217 in the smaller C_2_N sample. Early‐onset and late‐progression ages were 7‐8 years later for *p* ‐tau217 versus PiB‐SUVR using logistic‐curves.

**Conclusion:**

Late AD‐progression and covariate‐related shifts in progression were comparable across models, suggesting robustness to sigmoid choice. In contrast, estimates of early AD‐onset may be more dependent on functional form. While logistic‐sigmoids inherently increase monotonically, hyperbolas do not, here estimating a decline in initial amyloid trend. Future work may explore adding biologically informed constraints to flexible sigmoidal‐functions, and alternative Bayesian approaches, to optimize both model fit and estimation of early AD‐onset age.